# Effect of *Trichoderma viride* on insoluble phosphorus absorption ability and growth of *Melilotus officinalis*

**DOI:** 10.1038/s41598-023-39501-y

**Published:** 2023-07-31

**Authors:** Mingxia Song, Xinyu Wang, Hongwei Xu, Xiaofu Zhou, Chunsheng Mu

**Affiliations:** 1 Key Laboratory of Vegetation Ecology of the Ministry of Education, Institute of Grassland Science, Northeast Normal University, Changchun, China; 2 Tonghua Normal University, Tonghua, China; 3 Changchun Greening Management Center, Changchun, China; 4 Key Laboratory for Plant Resources Science and Green Production, Jilin Normal University, Siping, China

**Keywords:** Biotechnology, Plant sciences

## Abstract

Phosphorus (Pi) deficiency is a major factor of limiting plant growth. Using Phosphate-solubilizing microorganism (PSM) in synergy with plant root system which supply soluble Pi to plants is an environmentally friendly and efficient way to utilize Pi. *Trichoderma viride* (*T. viride*) is a biocontrol agent which able to solubilize soil nutrients, but little is known about its Pi solubilizing properties. The study used *T. viride* to inoculate *Melilotus officinalis* (*M. officinalis*) under different Pi levels and in order to investigate the effect on Pi absorption and growth of seedlings. The results found that *T. viride* could not only solubilizate insoluble inorganic Pi but also mineralize insoluble organic Pi. In addition, the ability of mineralization to insoluble organic Pi is more stronger. Under different Pi levels, inoculation of *T. viride* showed that promoted the growth of aboveground parts of seedlings and regulated the morphology of roots, thus increasing the dry weight of seedlings. The effect of *T. viride* on seedling growth was also reflected the increasing of chlorophyll fluorescence parameters and photosynthetic pigment content. Moreover, compared to the uninoculated treatments, inoculation of *T. viride* also enhanced Pi content in seedlings. Thus, the *T. viride* was a beneficial fungus for synergistic the plant Pi uptake and growth.

## Introduction

Pi is an essential plant macronutrient required for growth and development^1^, which plays many key roles in plant life such as synthesis of nucleotides, composition of membranes, photosynthesis, respiration, carbohydrate transport, etc^2^. The main form of Pi used by plants is ortho-phosphate, but it is very easy to form insoluble compounds with Fe^3+^, Al^3+^, Ca^2+^ and Mg^2+^ in terrestrial ecosystems, so its low availability often limits the growth of plants^3^. Pi limitation is the main productivity limiting factor in agricultural^4,5^ and grassland ecosystems^6^. Although the Pi deficiency in plants can be alleviated by applying soluble Pi fertilizer to plants, but the utilization rate of fertilizer is greatly reduced due to chemical fixation of Pi and agricultural runoff^7^. However, overuse of phosphate rock fertilizers can lead to eutrophication of water body and ultimately to environmental degradation. Some studies have shown that phosphate rock is a non-renewable resource, and phosphate reserves will be exhausted in 50–100 years^8,9^. Therefore, how plants use insoluble Pi in the soil is a hot issue at present.

Using PSMs as Pi biofertilizer has been reported to be an economical and environmentally friendly way to dissolve insoluble Pi in soils^10^. Phosphate-solubilizing microorganisms (PSMs) include phosphate-solubilizing bacteria (PSB) and phosphate-solubilizing fungi (PSF), normally PSF has higher phosphate-solubilizing activity^11^. In soil, PSB or PSF convert the insoluble Pi into soluble Pi for plant absorption and utilization by secreting organic acids and phosphatase^12^. PSMs have been shown to secrete phosphatases to hydrolyze organophosphorus in soil^13^. For example, the *Bacillus subtilis* strain KPS-11 could produce extracellular phytase to mineralize insoluble organophosphorus, which significantly promoted the absorption of insoluble organophosphorus in potatoes^14^. PSM is involved in solubilizing the insoluble inorganic Pi by producing organic acid e.g., gluconic, 2-ketogluconic acid, malic, lactic, acetic, citric, and succinic acid^15–17^. Some PSMs have two mechanisms at the same time, including Pi solubilization and Pi mineralization, but some PSMs have only one of them. For instance, Gomez-Ramirez et al.^18^ found that the *Bacillus* spp. strain IBUN-02724 had two mechanisms of Pi solubilization/mineralization at the same time, which not only was able to solubilize insoluble inorganic Pi from Ca_3_(PO_4_)_2_ and AlPO_4_, but also mineralize insoluble organic Pi from phytate.

PSMs are the main engine to promote the circulation of Pi between soil and plants^12,19^. In addition to its own Pi solubilizing characteristics, PSM can also regulate soil Pi uptake by plant roots through different mechanisms^20^. The interaction between PSM and plant can trigger the responsive mechanisms to control soil Pi flow to roots by regulating root exudation and root structure^21^. Moreover, PSM can affect the uptake and transport of soil Pi by plants through regulating the expression of plant Pi transporter protein genes^22^. Besides providing Pi for plant uptake, PSM promotes plant growth by producing beneficial metabolites^23,24^. In exchange, plants need to provide PSM with photosynthates for their growth^25^. However, a recent study by Clausing and Polle showed that some PSMs compete with plant roots for Pi in low Pi soils^26^. Moreover, Tian et al^27^ found that the addition of Pi fertilizer could inhibit the growth of some PSMs, such as *Bacillales* and *Pseudomonadales*. Thus, the application of PSM in agricultural production still needs a large number of experiments to screen and verify which is able to ensure the effect of increasing production.

Fungi of the genus *Trichoderma* belong to the phylum Ascomycota, subdivision Pezizomycotina, class Sordariomycetes, order Hypocreales and family Hipocreaceae^28^. *Trichoderma* spp. are common rhizosphere fungus which have been regarded as beneficial fungi due to the function of promoting the growth of plants and pathogen suppression ability^29^. Therefore, *Trichoderma* spp. are often used as biocontrol agents and biofertilizers to improve crop yields^30^. *Trichoderma* spp. have the function of promoting plant growth mainly because of the ability to dissolve nutrients from the soil, changing the root structure, secreting indoleacetic acid, cytokinin, gibberellin, gibberellins, and zeatin^31,32^. Recently, Zhang et al*.*^33^ observed that *Trichoderma* species could increase the activity of antioxidant enzyme in plants such as peroxidase, superoxide dismutase, and catalase, however, decrease the content of the hydrogen peroxide and the superoxide radical, which enhanced disease resistance of plants. Rudresh et al*.*^34^ reported that different species of *Trichoderma* were able to dissolve tricalcium phosphate (TCP) in vitro and in vivo, including *Trichoderma viride*, *Trichoderma virens* and *Trichoderma harzianum*. However, there have been few studies to conduct on the solubilization of insoluble Pi in soil by *T. viride* and the effect of interaction with plants on growth. In particular, studies on the promotion of insoluble organic Pi uptake to plants by *T. viride* are rarely reported. Therefore, it is necessary to clarify that *T. viride* has the property of solubilizing/mineralizing insoluble Pi. Here, we used *T. viride* to inoculate *M. officinalis* under insoluble Pi conditions. In the present study, we tried to answer the questions of whether *T. viride* dissolves/mineralizes insoluble Pi to supply *M. officinalis* with soluble Pi and the effect of *T. viride* on the physiological properties of *M. officinalis*.

## Materials and methods

### Cultivation of *T. viride*

The tested strain was *T. viride*, deposited in China General Microbiological Culture Collection Center (CGMCC; No.40034). The *T. viride* was placed on potato dextrose agar (PDA) culture medium and incubated at 28 °C in the dark for 7 d. The *T. viride* spores were flushed by sterile water which contained 0.01% Tween-80. The spore concentration was adjusted to 1 × 10^8^ cfu·mL^−1^ with a haemocytometer.

### Cultivation of seedlings

The seeds of *M. officinalis* were acquired from the Institute of Grassland Science of Northeast normal University, China. The seeds were soaked in 75% ethanol for 3 min, and followed by 2% NaOCl for 10 min with agitation. Then, sterilized seeds were germinated in plastic square pots (20 cm length × 15 cm width × 12 cm high) containing sterilized vermiculite as a substrate. Each pot contains 18 seedlings was poured 300 mL of 1/2 strength Hoagland nutrient solution every 3 days. The 1/2 strength Hoagland nutrient solution consisting of 2.5 mM KNO_3_, 2.5 mM Ca(NO_3_)_2_, 1.0 mM MgSO_4_·7H_2_O, 500 μM KH_2_PO_4_, 22.5 µM H_3_BO_3_, 5 µM MnCl_2_·4H_2_O, 0.4 µM ZnSO_4_·7H_2_O, 0.15 µM CuSO_4_·5H_2_O, 0.2 µM Na_2_MoO_4_·2H_2_O and 10 µM Fe-EDTA. The pH of the nutrient solution was 6.0. The 500 mL water was poured into the each pot every 5 days. Seedlings were grown for 30 days at 26 °C/22 °C in day/night temperature with a 16-h photoperiod, an irradiance of 480 µmol m^−2^ s^−1^, and relative humidity 65–70%. The growth conditions during the experimental period were the same as that in different Pi stess treatment experiment.

## Experimental design

**Figure 1:**
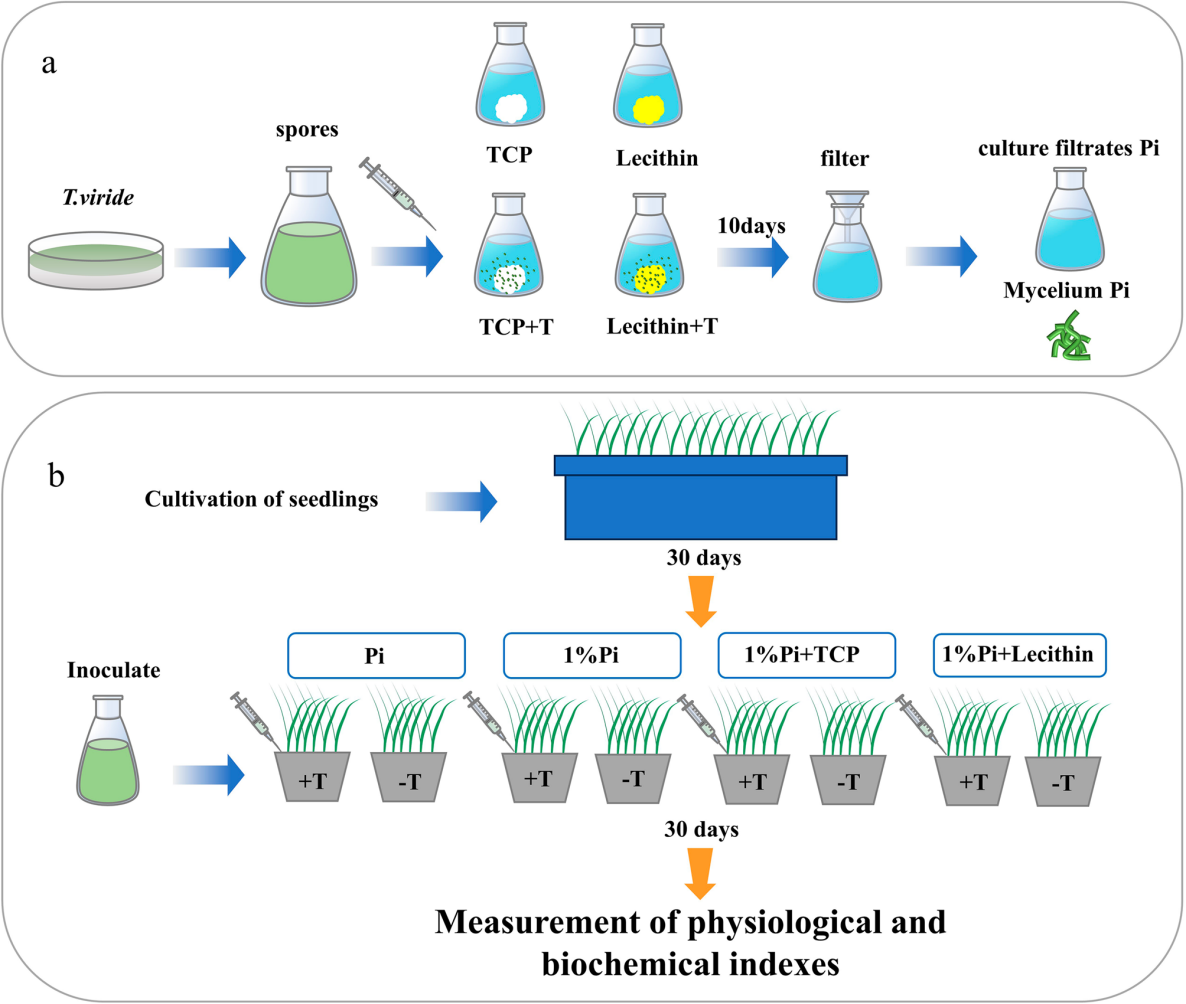
Experimental design flow chart.

The experiment was conducted using a two factor random block design with two factors, including different Pi forms and *T. viride*. The experiment designed eight treatments and each treatment had six replicates (Fig. 1b). The 1/2 strength Hoagland nutrient solution was used in this study containing two levels of Pi concentration, 500 μM KH_2_PO_4_ (Pi) and 5 μM KH_2_PO_4_ (1%Pi). The nutrient solution containing 5 μM KH_2_PO_4_ was prepared by substituting KCl for KH_2_PO_4_ so that the concentration of K in the nutrient solution was 0.5 mM for all treatments. Tricalcium phosphate (TCP) and egg yolk lecithin (Lecithin) was selected to be used as insoluble Pi. The applied treatments were Pi: irrigating seedlings with 1/2 strength Hoagland nutrient solution containing 500 μM KH_2_PO_4_; Pi + T: irrigating seedlings with 1/2 strength Hoagland nutrient solution containing 500 μM KH_2_PO_4_ and each seedling was inoculated with 5 mL of *T. viride* spores fluid; 1%Pi: irrigating seedlings with 1/2 strength Hoagland nutrient solution containing 5 μM KH_2_PO_4_; 1%Pi + T: irrigating seedlings with 1/2 strength Hoagland nutrient solution containing 5 μM KH_2_PO_4_ and each seedling was inoculated with 5 mL of *T. viride* spores fluid; 1%Pi + TCP: each pot containing TCP, and irrigating seedlings with 1/2 strength Hoagland nutrient solution containing 5 μM KH_2_PO_4_; 1%Pi + TCP + T: Each pot containing TCP, and irrigating seedlings with 1/2 strength Hoagland nutrient solution containing 5 μM KH_2_PO_4_, and each seedling was inoculated with 5 mL of *T. viride* spores fluid; 1%Pi + Lecithin: Each pot containing Lecithin, and irrigating seedlings with 1/2 strength Hoagland nutrient solution containing 5 μM KH_2_PO_4_; 1%Pi + Lecithin + T: each pot containing Lecithin, and irrigating seedlings with 1/2 strength Hoagland nutrient solution containing 5 μM KH_2_PO_4_, and each seedling was inoculated with 5 mL of *T. viride* spores fluid. The Pi element applied in the treatment of Pi was 68 mg. The total amount of Pi in insoluble Pi treatments were the same as that in the treatment of Pi. Every 30-day-old seedlings were transplanted into plastic pots (15 cm diameter × 12 cm high) for the different treatments which were poured 100 mL of 1/2 strength Hoagland nutrient solution evry 3 days to culture the plants for 30 days. Then, the plants were harvested for using in the physiological and biochemical assays.

### Determination of the ability to release insoluble Pi of *T. viride* and acidity in culture filtrates

Four treatments were set up with NBRIP (National Botanical Research Institute’s Phosphate) as liquid medium. TCP and Lecithin were selected as insoluble Pi sources. The treatment with only added insoluble Pi was the control group, and with added insoluble Pi and *T. viride* was the experimental group (Fig. 1a). For this assay, the *T. viride* spores were inoculated into 100 mL of NBRIP liquid medium in 250 mL conical flasks and pH was adjusted to 7.0. The concentration of inoculated spores was 1 × 10^8^ cfu·mL^−1^ and the proportion of inoculated spores was 2%. All treatments were incubated at 28 °C in the dark for 10 days. Then, the supernatants was tested for available Pi and mycelium was tested for total Pi using molybdenum blue method^36^. Culture filtrates was collected after centrifugation of 10-day-old and measured the pH value.

### Determination of growth indices

The plant height and leaf area was measured using a precision ruler. The formula for calculating leaf area was: leaf area (cm^2^) = leaf length × leaf width × 0.75^37^. Stem diameter was measured using a vernier caliper. Shoots and roots were harvested from each pot and then the roots were washed by water to remove the vermiculite. The fresh shoots and roots were dried at 105 °C for 30 min and after that the temperature was reduced to 70 °C for 10 h. Using a balance with an accuracy of 1/10,000 to measure the dry weight of shoots and roots. The fresh washed roots were scanned using a desktop scanner (EPSON Perfection V 700 Photo; Epson, America, Inc., USA). The resulting image was processed using WinRHIZO image analysis system (Win RHIZO 2012 b; Regent, Canada) to determine the morphological characteristics of the roots.

### Measurement of chlorophyll (Chl*a*) fluorescence parameters

The Chl*a* parameters of different treatments were monitored using a MAXI-Imaging PAM M-Series (Heinz Walz GmbH, Effeltrich, Germany). All treatments were kept in a dark adaptation period of 20 min before measurements. The chlorophyll fluorescence parameters were analyzed, such as Maximum efficiency of PSII photochemistry (Fv/Fm), effective PSII quantum yield (ΦPSII or Y(II)), non-photochemical quenching (NPQ), photochemical quenching (qP)^35^. In order to determine the incidence of the irradiance level on the PSII fluorescence emissions, rapid light curves (RLCs) composed of 16 steps, with durations at each photosynthetically active radiation (PAR) level of 20 s, were performed (0, 1, 20, 55, 110, 185, 280, 335, 395, 460, 530, 610, 700, 800, 925, 1075, 1250 µmol photons m^−2^ s^−1^). Draw a line chart of the fluorescence parameters varying with light intensity, such as Y(II), qP, NPQ, electron transport rate (ETR).

### Determination of chlorophyll and carotenoid content

The top fresh leaves were cut off from each sample, and weighed to 0.5 g for extracting Chlorophyll (Chl). The leaves were immersed into 95% (v/v) ethanol until complete bleaching. The concentration was determined by measuring extract absorbances at 470 nm, 649 nm, and 665 nm in a spectrophotometer (Hitachi U-3000; Hitachi, Ltd., Chiyoda, Tokyo, Japan). The Chl *a*, Chl *b*, and carotenoid (Car) was calculated using the following formulas^38^:

$$\begin{gathered} {\text{Chl }}a \, = { 13}.{\text{95A}}_{{{665}}} - {6}.{\text{88A}}_{{{649}}} \hfill \\ {\text{Chl }}b \, = { 24}.{\text{96A}}_{{{649}}} - {7}.{\text{32A}}_{{{665}}} \hfill \\ {\text{Car }} = \, ({1}000{\text{A}}_{{{47}0}} - {2}.0{\text{5 Chl }}a - {114}.{\text{8 Chl }}b)/{245} \hfill \\ \end{gathered}$$

### Determination of total Pi content in tissues

The shoots and roots of the samples were separated and separately dried at 105 °C for 30 min. Then the samples were dried at 65 ◦C for 10 h. The total Pi content of the shoots and roots was measured by the molybdenum blue method^36^.

### Data analysis and statistics

All data from the vitro studies of *T. viride* and pot experiments from six replicates were analyzed statistically by analysis of variance (ANOVA). We quantified the effects of different Pi treatments on the dry weight and Pi content with or without *T. viride* inoculation using the two-way ANOVA. Comparison of means were made by Student–Newman–Keuls test(S–N–K) (p < 0.05). All statistical analyses were performed with the SPSS statistical software 26.0, and figures were drawn with OriginPro 2021.

### Statement

Our research is in line with local and national guidelines.

## Results

### The ability to release insoluble Pi of *T. viride* and acidity in culture filtrates

The *T. viride* showed Pi solubilization activity in NBRIP medium along with a subsequent decrease in pH (Table 1). The content of soluble Pi in the culture filtrates treated with TCP and *T. viride* was 394.75% higher than that treated with TCP. While, the content of soluble Pi in the culture filtrates treated with Lecithin and *T. viride* was 29.95% higher than that treated with Lecithin. The mycelium Pi in the treatment of TCP with *T. viride* was 14.90 mg·L^−1^ and which in the treatment of Lecithin was 48.94 mg·L^−1^. The pH value in the treatment of TCP with *T. viride* decreased 29.29% than that without *T. viride*. Similarly, the pH value in the treatment of Lecithin with *T. viride* decreased 41.26% than that without *T. viride*.

**Table 1:** Pi content of culture filtrates and mycelium and pH value of medium under diferent treatments.

Treatment	culture filtrates Pi (mg·L^-1^)	Mycelium Pi (mg·kg^-1^)	Ph
TCP	0.24 ± 0.16c	0.00 ± 0.00c	6.93 ± 0.01a
TCP + T	94.98 ± 3.10a	14.90 ± 0.12b	4.90 ± 0.06b
Lecithin	1.87 ± 0.14c	0.00 ± 0.00c	4.46 ± 0.03c
Lecithin + T	57.88 ± 0.25b	48.94 ± 0.11a	2.62 ± 0.14d

Values are the means ± S.E. (n = 6) based on analyses by one-way ANOVAs followed by S–N–K test. Different letters indicate significant difference (P < 0.05).

*TCP* tricalcium phosphate, *Lecithin* egg yolk lecithin, *T* *T. viride*.

### Growth indices

Pi content and *T. viride* was very important for seedling growth. The growth indices of the treatments with *T. viride* were higher than those without *T. viride* (Table 2). The plant height in the 1%Pi + Lecithin + T treatment was 2.42% higher than the 1% Pi + TCP + T treatment. However, plant height of the 1%Pi treatment was the lowest among other treatments. Plant height in the 1%Pi + T treatment was on par with the 1%Pi treatment and the Pi + T treatment was pretty much the same as the Pi treatment. The stem diameter of the 1%Pi + Lecithin + T treatment was similar to the 1% Pi + TCP + T treatment, but were 10.14% and 22.76% higher than which treatments without *T. viride*, respectively. There was no significant difference between the Pi treatment and the Pi + T treatment in leaf area. The leaf area in the 1%Pi + Lecithin + T treatment was 5.67% higer than the 1% Pi + TCP + T treatment. The shoot dry weight of the Pi + T treatment was the highest than the other treatments. The shoot dry weight of the 1%Pi + Lecithin + T treatment was similar to the 1% Pi + TCP + T treatment, but were 54.02% and 34.30% higher than which treatments without *T. viride*, respectively. The root dry weight in the Pi treatment and the Pi + T treatment were similar and higher than the other treatments. The root dry weight of the 1% Pi + Lecithin + T was 19.23% higher than the 1%Pi + TCP + T treatment. There was a significant positive correlation between parameters of aboveground growth and total Pi content (Fig. 5).

**Table 2:** Plant height and stem diameter and leaf area and shoot/root dry weight under different treatments.

Treatment	Plant height (cm)	Stem diameter (mm)	Leaf area (cm^2^)	Shoot dry weight (g)	Root dry weight (g)
Pi	21.97 ± 2.69a	1.55 ± 0.04a	3.82 ± 0.07a	0.422 ± 0.005b	0.389 ± 0.004a
Pi + T	22.13 ± 1.98a	1.59 ± 0.03a	3.91 ± 0.07a	0.494 ± 0.006a	0.382 ± 0.002a
1%Pi	17.83 ± 1.08c	1.09 ± 0.08d	1.46 ± 0.36e	0.195 ± 0.002f	0.128 ± 0.004g
1%Pi + T	17.92 ± 2.10c	1.19 ± 0.09c	1.99 ± 0.28d	0.230 ± 0.020e	0.139 ± 0.002f
1%Pi + TCP	18.87 ± 0.52bc	1.23 ± 0.10c	2.42 ± 0.26c	0.242 ± 0.003de	0.171 ± 0.003e
1%Pi + TCP + T	21.07 ± 0.99ab	1.51 ± 0.01a	3.35 ± 0.16b	0.325 ± 0.003c	0.208 ± 0.003c
1%Pi + lecithin	18.48 ± 3.01bc	1.38 ± 0.09b	2.56 ± 0.55c	0.261 ± 0.004d	0.189 ± 0.002d
1%Pi + lecithin + T	21.58 ± 0.70a	1.52 ± 0.04a	3.54 ± 0.28ab	0.402 ± 0.005c	0.248 ± 0.014b

Values are the means ± S.E. (n = 6) based on analyses by one-way ANOVAs followed by S–N–K test. Different letters indicate significant difference (P < 0.05).

The root morphological parameters of inoculated *T. viride* were higher than those of uninoculated treatments (Table 3). The root morphological parameters of the 1%Pi + Lecithin + T treatment were higher than the 1%Pi + Lecithin treatment. The root morphological parameters of the 1%Pi + TCP + T treatment were higher than the 1%Pi + TCP treatment. The root morphological parameters of the Pi + T treatment were higher than the Pi treatment, and as well as the 1%Pi + T treatment which were higher than the 1%Pi treatment. There was a significant positive correlation between parameters of root morphology and total Pi content (Fig. 5).

**Table 3:** Root morphological change under diferent Pi treatments.

Treatments	Root length (cm)	Root volume (cm^3^)	Root surface area (cm^2^)	Root diameter (mm)	Number of root tips	Number of crossings	Number of forks
Pi	454.53 ± 19.46a	2.38 ± 0.34ab	15.48 ± 0.80ab	0.52 ± 0.07ab	1642.50 ± 304.75b	1629.00 ± 223.92b	5375.00 ± 376.11a
Pi + T	469.75 ± 13.59a	2.74 ± 0.55a	16.03 ± 0.57a	0.55 ± 0.06a	1854.33 ± 145.67a	2119.67 ± 418.25a	5048.67 ± 551.52ab
1% Pi	308.83 ± 44.88c	1.02 ± 0.19d	11.40 ± 1.25d	0.36 ± 0.02c	677.33 ± 122.52e	804.50 ± 85.74d	3681.50 ± 622.36d
1% Pi + T	333.63 ± 32.72c	1.44 ± 0.32c	12.73 ± 0.82c	0.39 ± 0.02c	836.50 ± 95.70de	820.83 ± 89.88d	3781.17 ± 462.17cd
1% Pi + TCP	321.21 ± 43.18c	1.92 ± 0.49b	12.98 ± 0.65c	0.39 ± 0.03c	852.50 ± 80.48de	874.50 ± 70.40d	4369.17 ± 470.95bc
1% Pi + TCP + T	385.45 ± 12.41b	2.13 ± 0.20b	14.54 ± 0.51b	0.48 ± 0.03b	1288.83 ± 164.05c	1346.33 ± 79.14c	4665.67 ± 435.93ab
1% Pi + lecithin	336.22 ± 14.90c	2.09 ± 0.13b	13.23 ± 1.13c	0.41 ± 0.03c	974.00 ± 168.05d	959.17 ± 75.49d	4433.83 ± 480.50bc
1%Pi + lecithin + T	470.25 ± 13.39a	2.28 ± 0.30ab	15.81 ± 0.63a	0.48 ± 0.03b	1613.17 ± 179.99b	1452.67 ± 50.03bc	4983.17 ± 446.05ab

Values are the means ± S.E. (n = 6) based on analyses by one-way ANOVAs followed by S–N-K test. Different letters indicate significant difference (P < 0.05).

### Parameters of Chlorophyll *a* fluorescence

**Figure 2:**
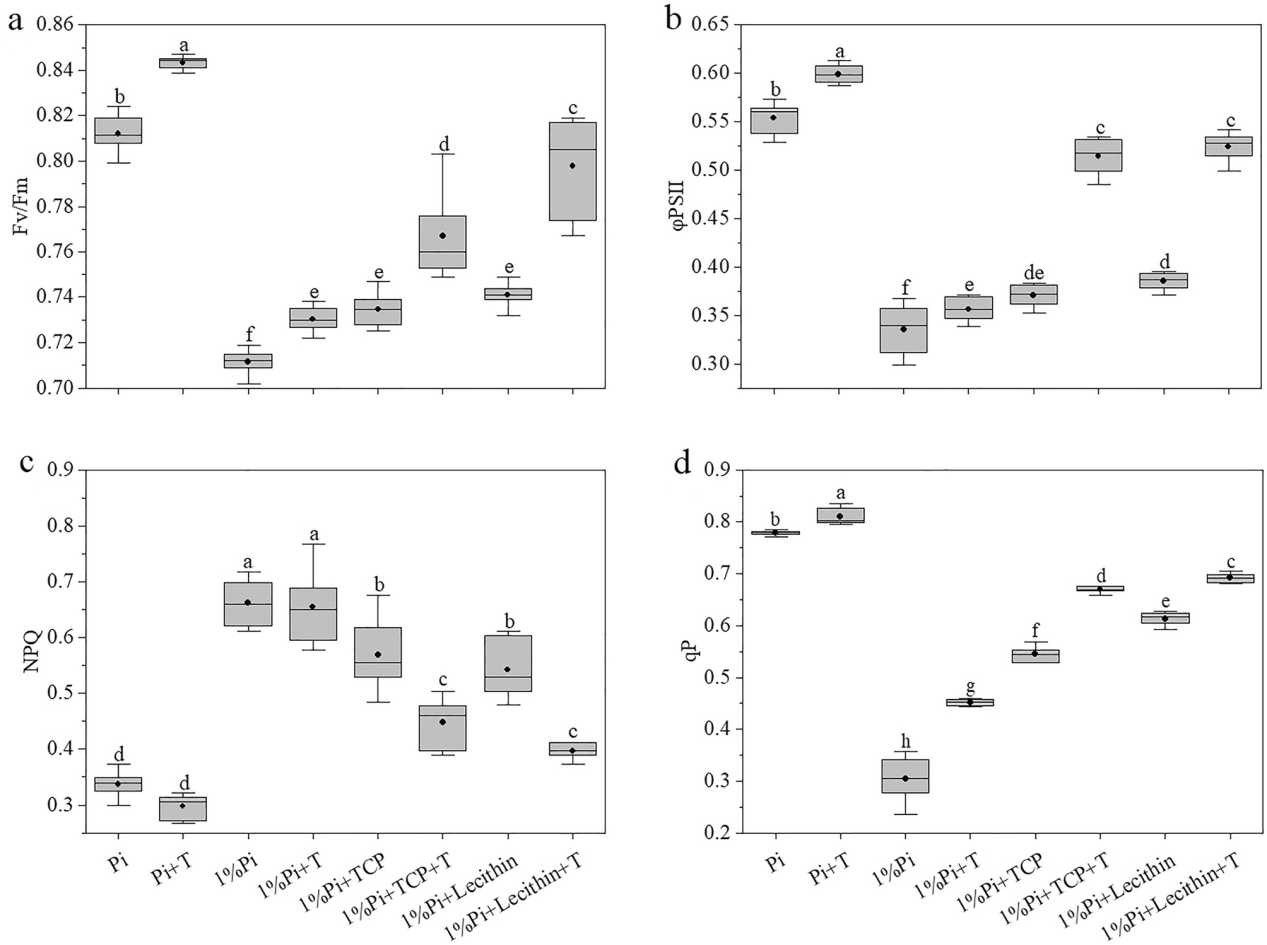
Comparisons of chlorophyll fluorescence parameters: (**a**) maximum quantum yield of PSII (Fv/Fm), (**b**) effective quantum yield of PSII (ϕPSII), (**c**) nonphotochemical quenching (NPQ), (**d**) coefficient of photochemical quenching(qP). Values are the means ± S.E. (n = 6) based on analyses by one-way ANOVAs followed by S–N–K test. Different letters indicate significant difference (P < 0.05).

Compared to the non-inoculated treatment, the *T. viride* inoculation increased Fv/Fm, ϕPSII and qP, but decreased NPQ (Fig. 2). The results showed that the highest Fv/Fm was the Pi + T treatment, and the lowest was the 1%Pi treatment (Fig. 2a). In insoluble-Pi treatments which inoculated *T. viride*, Fv/Fm of the 1%Pi + Lecithin + T treatment was higher than the 1%Pi + TCP + T treatment. In low Pi treatments, Fv/Fm of the 1%Pi + T treatment was higher than the 1%Pi treatment. The results showed that the highest ϕPSII was the Pi + T treatment, and the lowest was the 1%Pi treatment (Fig. 2b). In insoluble-Pi treatments which inoculated *T. viride*, there was no significant difference of ϕPSII between the 1%Pi + Lecithin + T treatment and the 1%Pi + TCP + T treatment. In low Pi treatments, ϕPSII of the 1%Pi + T treatment was higher than the 1%Pi treatment. The results showed that the highest qP was the Pi + T treatment, and the lowest was the 1%Pi treatment (Fig. 2c). In insoluble Pi treatments which inoculated *T. viride*, qP of the 1%Pi + Lecithin + T treatment was higher than the 1%Pi + TCP + T treatment. In low Pi treatments, qP of the 1%Pi + T treatment was higher than the 1%Pi treatment. From the resaults, we found that the highest NPQ were the 1%Pi treatment and the 1%Pi + T treatment, the lowest were the Pi treatment and the Pi + T treatment (Fig. 2d). In insoluble-Pi treatments which inoculated *T. viride*, NPQ of the 1%Pi + Lecithin + T treatment was lower than the 1%Pi + Lecithin treatment and the 1%Pi + TCP + T treatment was lower than the 1%Pi + TCP treatment. However, there was no significant difference of NPQ between the 1%Pi + Lecithin + T treatment and the 1%Pi + TCP + T treatment and the same as the 1%Pi + Lecithin treatment and the 1%Pi + TCP treatment. In low Pi treatments, there was no significant difference of NPQ between the 1%Pi treatment and the 1%Pi + T treatment. There was a significant positive correlation between parameters of chlorophyll *a* fluorescence and shoot total Pi content (Fig. 5).

**Figure 3:**
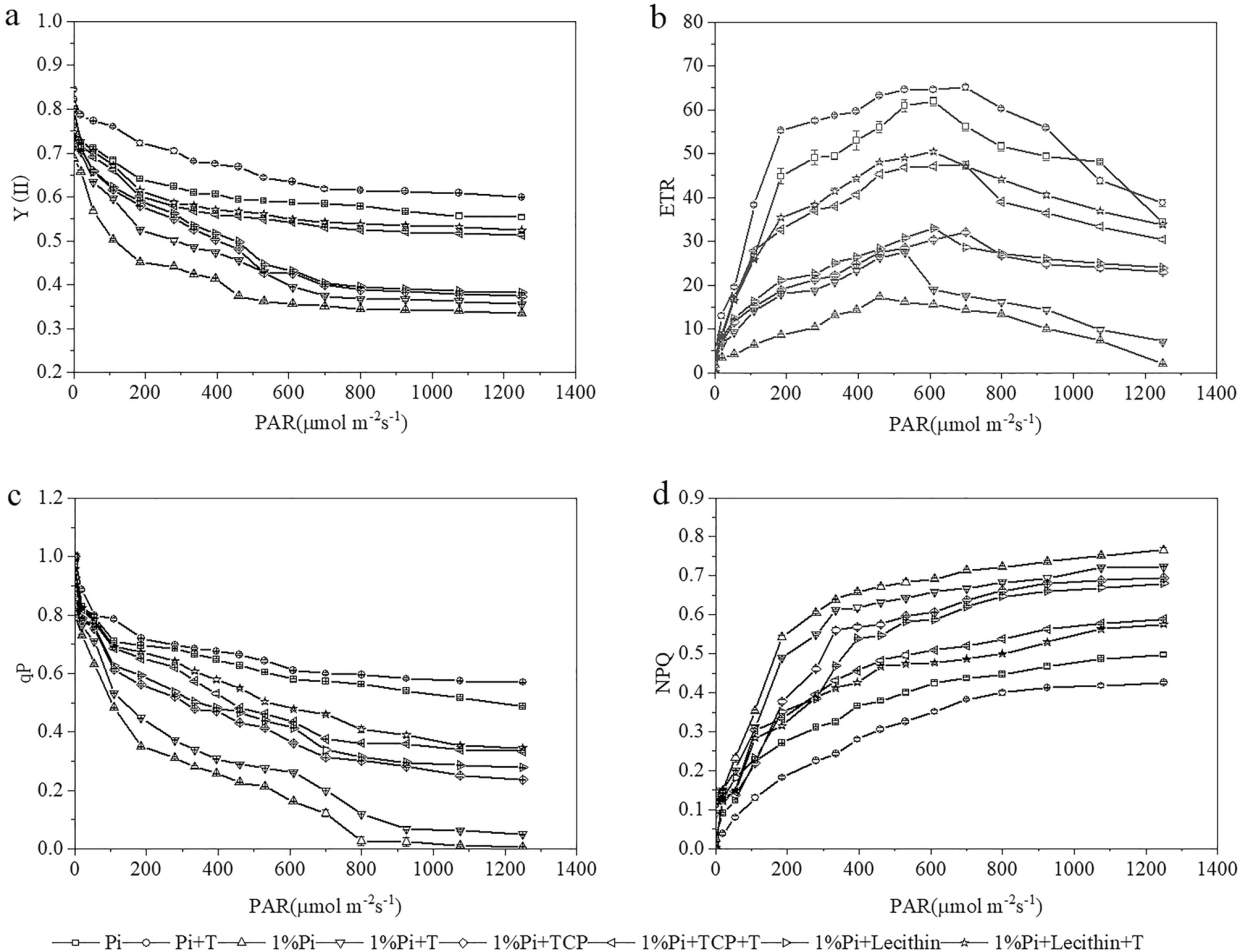
Chlorophyll fluorescence parameters as a function of PAR: (**a**) plots of Y (II) vs. PAR, (**b**) photosynthetic electron transfer rates (ETR) vs. PAR, (**c**) qP vs. PAR, (**d**) NPQ vs. PAR. Values are the means ± S.E. (n = 6) based on analyses by one-way ANOVAs followed by S–N–K test. Different letters indicate significant difference (P < 0.05).

We also analyzed the light-response curves of the Y (II), ETR, qP, and NPQ of PSII under the condition of the *T. viride* inoculation or non-inoculation in deffrent Pi supply (Fig. 3). Y(II) reduction of the inoculated *T. viride* treatment was more slowly than uninoculated *T. viride* treatment under light intensity (PAR < 500 µmol·m^−2^·s^−1^) (Fig. 3a). When light intensities > 500 µmol·m^−2^·s^−1^, the production of light quantum production was inhibited. ETR increasing of the inoculated *T. viride* treatment was more faster than uninoculated *T. viride* treatment under a certain range of light intensity (PAR < 500 µmol·m^−2^·s^−1^), subsequently reached plateaued (Fig. 3b). qP of the *T. viride* inoculation treatment decreased slowly compared with the *T. viride* uninoculation treatment under light intensity (PAR < 500 µmol·m^−2^·s^−1^) (Fig. 3c). NPQ of the *T. viride* uninoculation treatment rose sharply relative to the *T. viride* inoculation treatment under light intensity (PAR < 500 µmol·m^−2^·s^−1^) (Fig. 3d). Excess excitation energy was dissipated as heat energy under the condition of low Pi and insoluble Pi. Therefore, inoculation with *T. viride* alleviated PSII impairment to some extent.

### The content of chlorophyll, carotenoid and Chl *a*/Chl* b* ratio

The content of chlorophyll *a*, chlorophyll *b* and carotenoid content in the treatment with *T. viride* was significantly higher than that for the treatment without *T. viride*. There were no significant differences in Chl *a*/Chl *b* ratios among all treatments (Table 4). The chlorophyll *a* content, chlorophyll *b* content, carotenoid content and Chl *a*/Chl *b* ratio in Pi + T was the highest than other treatments. In the Pi + T treatment, the chlorophyll *a* content was 14.69% higher than the Pi, but there was no significant difference in chlorophyll *b* content, carotenoid content, Chl *a*/Chl *b* ratio between the Pi + T treatment and the Pi treatment. The chlorophyll a content of the 1%Pi + Lecithin + T treatment was 41.35% higher than the 1%Pi + Lecithin treatment and the chlorophyll *b* content of the 1%Pi + Lecithin + T treatment was 11.94% higher than the 1%Pi + Lecithin treatment. However, there was no significant difference in carotenoid content, Chl *a*/Chl *b* ratio between the 1%Pi + Lecithin + T treatment and the 1%Pi + Lecithin treatment. The chlorophyll *a* content of the 1%Pi + TCP + T treatment was 27.62% higher than the 1%Pi + TCP treatment and the chlorophyll *b* content of the 1%Pi + TCP + T treatment was 13.85% higher than the 1%Pi + TCP treatment. However, there was no significant difference in carotenoid content, Chl *a*/Chl *b* ratio between the 1%Pi + Lecithin + T treatment and the 1%Pi + Lecithin treatment. There was no significant difference in Photosynthetic pigment parameters between the 1%Pi + T treatment and the 1%Pi treatment. photosynthetic pigment was positively correlated with aboveground growth Indices, chlorophyll *a* fluorescence except NPQ, and the correlation between Chl *a*/Chl *b* ratio and other parameters was not obvious (Fig. 5).

**Table 4:** Chlorophyll content and carotenoid content and the Chl *a*/Chl *b* ratios.

Treatment	Chlorophyll *a* content (mg·g^−1^ FW)	Chlorophyll *b* content (mg·g^−1^ FW)	Carotenoid content (mg·g^−1^ FW)	Chl *a*/Chl *b*
Pi	1.77 ± 0.04b	1.03 ± 0.03a	0.33 ± 0.07a	1.73 ± 0.06a
Pi + T	2.03 ± 0.02a	1.07 ± 0.06a	0.36 ± 0.05a	1.93 ± 0.10a
1%Pi	0.91 ± 0.11d	0.51 ± 0.02d	0.11 ± 0.01b	1.83 ± 0.26a
1%Pi + T	0.96 ± 0.06d	0.52 ± 0.01d	0.12 ± 0.01b	1.88 ± 0.15a
1%Pi + TCP	1.05 ± 0.11d	0.65 ± 0.02cd	0.16 ± 0.01b	1.63 ± 0.19a
1%Pi + TCP + T	1.34 ± 0.06c	0.74 ± 0.04bc	0.21 ± 0.01b	1.83 ± 0.05a
1%Pi + lecithin	1.04 ± 0.04d	0.67 ± 0.04cd	0.17 ± 0.01b	1.61 ± 0.18a
1%Pi + lecithin + T	1.47 ± 0.06c	0.75 ± 0.03b	0.22 ± 0.02b	2.10 ± 0.57a

Values are the means ± S.E. (n = 6) based on analyses by one-way ANOVAs followed by S–N–K test. Different letters indicate significant difference (P < 0.05).

### The content of Pi in shoot and root

**Figure 4:**
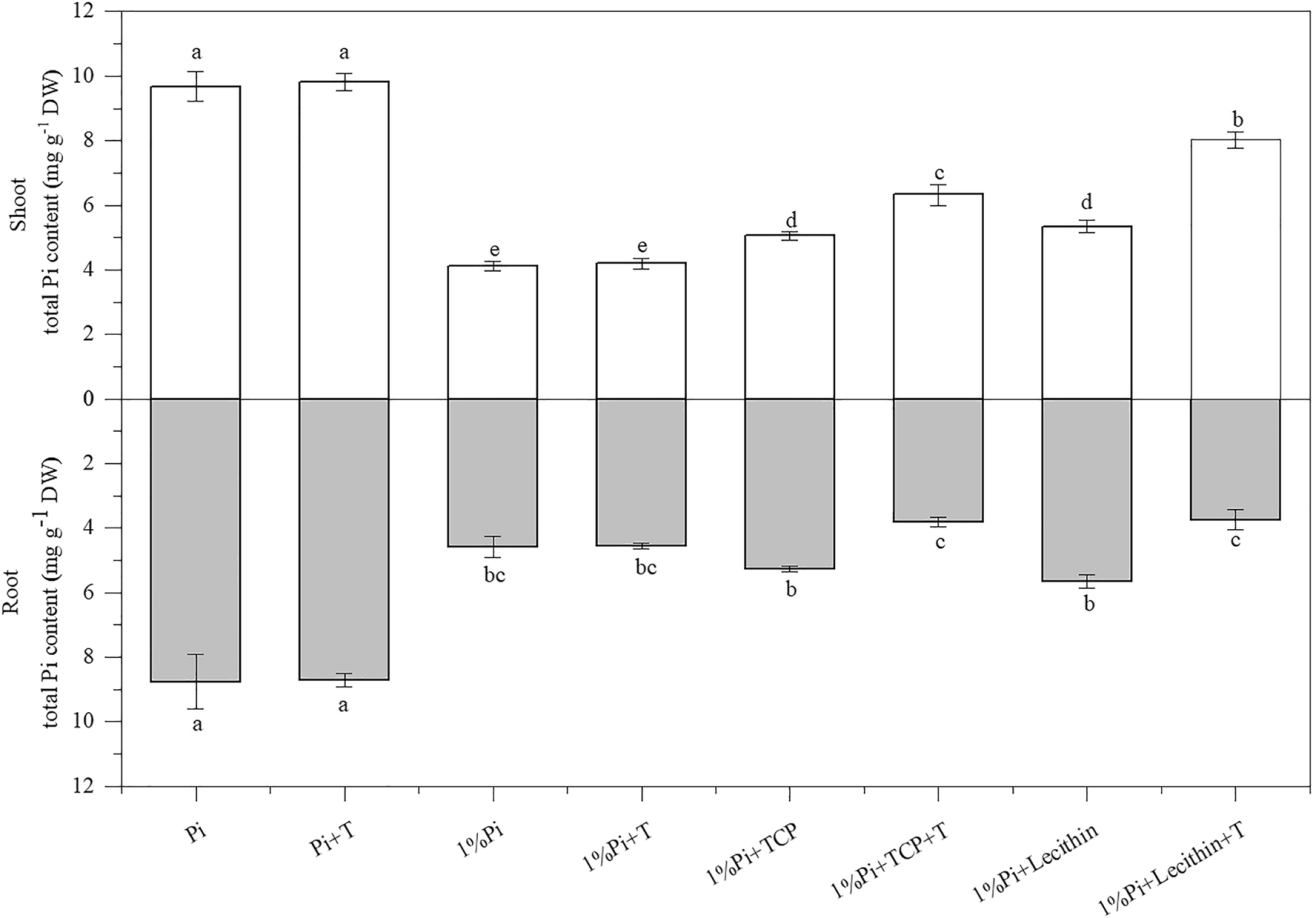
Shoot and Root Pi content. Values are the means ± S.E. (n = 6) based on analyses by one-way ANOVAs followed by S–N–K test. Different letters indicate significant difference (P < 0.05).

**Figure 5:**
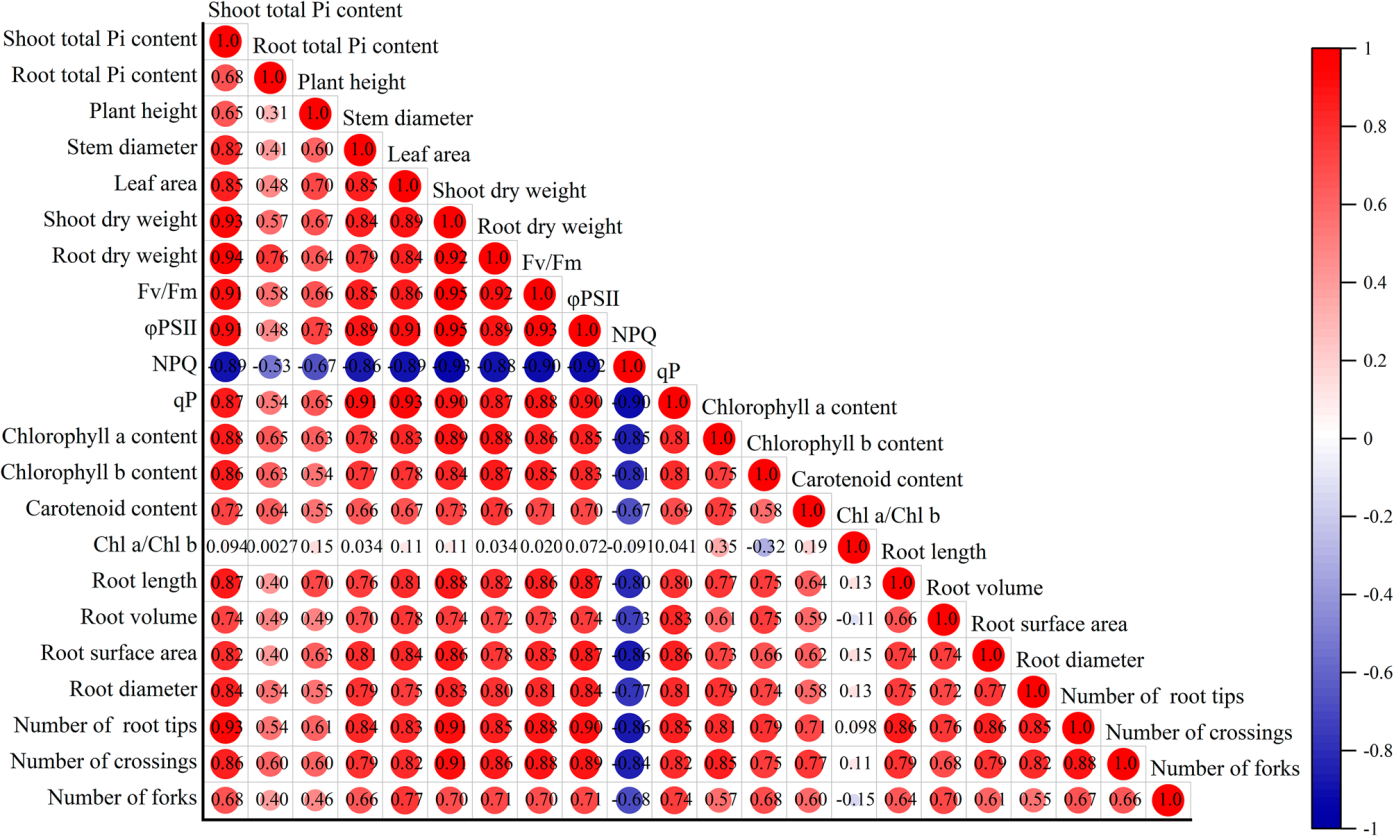
Correlation analysis of physiological and biochemical indexes.

Compared to the non-inoculated treatment, the *T. viride* inoculation increased the content of Pi in shoot and root. But there was no difference between the Pi treatment and the the Pi + T treatment, and which was no difference between the 1%Pi treatment and the the 1%Pi + T treatment (Fig. 4). On the other hand, plants grown with *T. viride* under insoluble Pi conditions were more easier to shift Pi from below-ground to above-ground tissues than the teatments without *T. viride*. In the 1%Pi + TCP + T treatment, the shoot Pi content was 65.46% higher than the root Pi content. In the 1%Pi + Lecithin + T treatment, the shoot Pi content was 114.43% higher than the root Pi content. The shoot Pi content was positively correlated with parameters of aboveground growth, root morphology, photosynthetic pigment, parameters of chlorophyll a fluorescence except NPQ (Fig. 5). The root Pi content was positively correlated with root dry weight, root morphology, but negatively correlated with NPQ (Fig. 5).

## Discussion

In recent years, there has been an growing interest in PSM. The use of PSM is an effective way to dissolve insoluble Pi in the soil and thus supply it to plants for uptake. At the same time, it had a beneficial effect on the growth and development of plants. In agricultural production, the application of PSM to the soil could reduce the use of chemical fertilizer and gradually replace the dominant position of chemical fertilizer^39,40^. Therefore, the present study evaluated the role of *T. viride* in Pi solubilization and in promoting the growth of *M. officinalis*.

The results showed that the *T. viride* could dissolve TCP and lecithin and reduce the pH value in the culture medium (Table 1). This might be because the PSM can dissolve insoluble Pi by releasing protons and producing organic acids to reduce the surrounding pH^41,42^. However, dissolving Pi is not a simple phenomenon, and each kind of PSM can use a variety of mechanisms to dissolve insoluble Pi. Therefore, it is necessary to further study the Pi solubilization mechanism of *T. viride*.

Inoculation of PSM has been reported to not only dissolve insoluble Pi in soil, but also promote plant growth ^43^. Our results showed that the growth indices of *M. officinalis* inoculated with *T. viride* were higher than the treatments without *T. viride* when insoluble Pi was used as Pi source (Table 2). The choices of Pi source and the addition of *T. viride* had a very strong effect on the dry weight of *M. officinalis*. This finding is consistent with previous work by Abdenaceur et al., who found that *Trichoderma* spp. secrete plant growth-promoting hormones, such as IAA ^44^. Root is an important organ for plants to absorb nutrients, which can sense the changes of nutrient concentration in soil, thus influencing changes in the root morphology of plants^45^. A study revealed that mung bean inoculated with PSB (*Pseudomonas* spp.) could increase root length and dry weight^46^. Our study also confirmed that the root morphological parameters of the treatment inoculated with *T. viride* were better than those without inoculation (Table 3). However, due to the presence of *T. viride*, *M. officinalis* changes its resource allocation strategy and is able to allocate more resources to above-ground growth and facilitate Pi uptake by the plant, allowing plants to have lower investment into below-ground biomass, and higher benefit for above-ground biomass (Table 3 and Fig. 4). Chlorophyll fluorescence parameters can characterize photosynthetic ability and energy conversion efficiency^47^. A study reported that under salt stress, inoculation of AMF or PSF increased the nutrient absorption of beach plums, and improved the parameters of chlorophyll fluorescence, such as Fv/Fm, qP and ϕPSII values, but NPQ values remained unchanged or decreased compared with the control^48^. Our results showed that the values of Fv/Fm, qP and ϕPSII of *M. officinalis* inoculated with *T. viride* under insoluble Pi stress were higher than those of the treatments without *T. viride*, while the values of NPQ were lower than the treatments without *T. viride* (Fig. 2). Moreover, the Y (II) and qP light-response curves of *M. officinalis* inoculated with *T. viride* decreased slowly compared with that without inoculation (Fig. 3). The ETR light-response curves of *M. officinalis* inoculated with *T. viride* increased faster than that without inoculation. The NPQ light-response curves of *M. officinalis* inoculated with *T. viride* rise more slowly than that without inoculation. Our study suggested that *M. officinalis* inoculated with *T. viride* could increase the efficiency of excitation energy capture by leaf chloroplasts and increase the photochemical capacity of PSII. The content of plant photosynthetic pigment shows the degree of plant stress and can be used as an indicator to evaluate the physiological status of plants^49^. Liu et al*.*^50^ found that the chlorophyll content of alfalfa inoculated with PSB was higher than that without inoculation. Similar to this study, the treatment inoculated with *T. viride* had higher Chlorophyll content and Carotenoid content and the Chl *a*/Chl *b* ratios than the uninoculated treatment (Table 4).

Qi et al*.*^51^ found that AMF were able to promote *Solidago canadensis* absorb more Pi in insoluble Pi conditions. A study reported that when phosphate rock was used as Pi source, *Trichoderma* spp. could dissolve phosphate rock and enhance the Pi content of chickpea shoots and roots, such as *T. viride*, *T. virens*, *T. virens*^34^. Our results also proved this point, the content of Pi in shoots and roots of *M. officinalis* inoculated with *T. viride* was higher than that wuthout inoculation (Fig. 4). *T. viride* and Pi source are the main factors, which affecting the Pi uptake and biomass of *M. officinalis*. Moreover, when insoluble Pi was used as Pi source, the Pi content of *M. officinalis* inoculated with *T. viride* was more distributed to the stem and supplied to the aboveground part of the plant. However, the *M. officinalis* without *T. viride* had more Pi content in the root under the condition of insoluble Pi. This may be due to the fact that the root system is the main organ for plants to absorb nutrients and plants do not need to expend more resources to invest in root growth under the condition of sufficient Pi.

## Conclusions

This study provides new evidence that *T. viride* can dissolve insoluble inorganic phosphorus and insoluble organic Pi. In addition to providing soluble phosphorus, *T. viride* also promotes plant growth of *M. officinalis* by improving root morphology and regulating plant photosynthesis. This research lays a foundation for recommending *T. viride* as a biological fertilizer and reduces environmental pollution caused by chemical fertilizer.

## Data Availability

The relevant experimental data are made available in Figshare. https://doi.org/10.6084/m9.figshare.22297105.
